# Coral-Associated Actinobacteria: Diversity, Abundance, and Biotechnological Potentials

**DOI:** 10.3389/fmicb.2016.00204

**Published:** 2016-02-29

**Authors:** Huda M. Mahmoud, Aisha A. Kalendar

**Affiliations:** Faculty of Science, Department of Biological Sciences, Kuwait UniversitySafat, Kuwait

**Keywords:** culturable coral-associated Actinobacteria, Arabian Gulf, antimicrobial ability, temporal and spatial variation, *Platygyra daedalea*

## Abstract

Marine Actinobacteria, particularly coral-associated Actinobacteria, have attracted attention recently. In this study, the abundance and diversity of Actinobacteria associated with three types of coral thriving in a thermally stressed coral reef system north of the Arabian Gulf were investigated. *Coscinaraea columna*, *Platygyra daedalea* and *Porites harrisoni* have been found to harbor equivalent numbers of culturable Actinobacteria in their tissues but not in their mucus. However, different culturable actinobacterial communities have been found to be associated with different coral hosts. Differences in the abundance and diversity of Actinobacteria were detected between the mucus and tissue of the same coral host. In addition, temporal and spatial variations in the abundance and diversity of the cultivable actinobacterial communities were detected. In total, 19 different actinobacterial genera, namely *Micrococcus*, *Brachybacterium*, *Brevibacterium*, *Streptomyces*, *Micromonospora*, *Renibacterium*, *Nocardia*, *Microbacterium*, *Dietzia*, *Cellulomonas*, *Ornithinimicrobium*, *Rhodococcus*, *Agrococcus*, *Kineococcus*, *Dermacoccus*, *Devriesea*, *Kocuria*, *Marmoricola*, and *Arthrobacter*, were isolated from the coral tissue and mucus samples. Furthermore, 82 isolates related to *Micromonospora*, *Brachybacterium*, *Nocardia*, *Micrococcus*, *Arthrobacter*, *Rhodococcus*, and *Streptomyces* showed antimicrobial activities against representative Gram-positive and/or Gram-negative bacteria. Even though *Brevibacterium* and *Kocuria* were the most dominant actinobacterial isolates, they failed to show any antimicrobial activity, whereas less dominant genera, such as *Streptomyces*, did show antimicrobial activity. Focusing on the diversity of coral-associated Actinobacteria may help to understand how corals thrive under harsh environmental conditions and may lead to the discovery of novel antimicrobial metabolites with potential biotechnological applications.

## Introduction

The marine environment is currently recognized as the largest potential source of new actinobacterial species because more than 70% of the planet is covered by oceans (Lam, 2006). At present, the discovery of rare or novel marine Actinobacteria has become a major focus in the search for the next generation of pharmaceutical agents (Bull et al., 2000). Marine Actinobacteria are expected to differ in their characteristics from their terrestrial counterparts and may produce new bioactive compounds (Manivasagan et al., 2013, 2014). In the literature, it is becoming evident that marine habitats are an abundant and novel source of Actinobacteria for new natural products because 716 new marine compounds were described in the Antibiotics Literature Database in 2004 (Blunt et al., 2006) and an additional 812 compounds were added in 2005 (Blunt et al., 2007). Culture-dependent and culture-independent molecular approaches have shown that marine Actinobacteria inhabit different marine habitats, including coastal and intertidal regions, marine sediments, seaweeds, fish, shrimps, mollusks and mangroves. Each of these environments has been found to harbor different members of Actinobacteria, some of which have antimicrobial activities (Mincer et al., 2002; Piza et al., 2004; Webster et al., 2004; Sivakumar et al., 2007).

Among marine systems, very little is known about actinobacterial diversity in coral reef systems. Corals, the most important members of the coral reefs, harbor abundant prokaryotic communities, including both Bacteria and Archaea (Rohwer et al., 2002) that inhabit coral mucus (Ducklow and Mitchell, 1979; Paul et al., 1986; Ritchie and Smith, 1997, 2004; Lampert et al., 2006), the tissue surface (Frias-Lopez et al., 2002; Bourne and Munn, 2005), the coral calcium carbonate skeleton and coral tissue (Williams et al., 1987; Shashar et al., 1994; Kushmaro et al., 1996; Banin et al., 2001). Lampert et al. (2006) have investigated the cultured bacteria associated with the mucus of the Red Sea coral *Fungia scutaria* and have found it to harbor different bacterial members, 23% of which were Actinobacteria. In addition, the mucus of *Fungia granulose* from the Red Sea (Kooperman et al., 2007), *Porites astreoides* from Bocas del Toro, Panama (Wegley et al., 2007), *Montipora capitata*, *Porites compressa* and *Porites lobata* (Ritchie and Lewis, 2005) has been found to harbor actinobacterial members. Furthermore, the culture-independent studies conducted by Yakimov et al. (2006) and Penn et al. (2006) have proven the presence of Actinobacteria in the deep-water corals of the Mediterranean Sea and the Gulf of Alaska Seamounts, respectively. Studies showed that healthy corals harbor larger numbers of Actinobacteria than their diseased counterparts (Frias-Lopez et al., 2002; de Castro et al., 2010). The capability of Actinobacteria to secrete a wide range of secondary metabolites against other microbes (Caundliffe, 2006; Piskorska et al., 2007) and their ability to fix nitrogen are expected to explain their dominance in healthy corals (Rohwer et al., 2002). Nithyanand et al. (2010, 2011) have found Actinobacteria associated with the branched coral *Acropora digitifera* from the Gulf of Mannar, India, with antibiotic activity against Gram-positive and Gram-negative bacteria. All of these studies investigated Actinobacteria associated with corals from tropical water bodies, but no information is available for thermally stressed corals, which are a potential reservoir for novel Actinobacteria species.

The Arabian Gulf is known as one of the hottest water bodies in the world (Kinsman, 1964; Sheppard et al., 1992), and corals of the Arabian Gulf are considered to be unique because they are able to survive extreme fluctuations in temperature (Riegl and Purkis, 2012). Corals usually perish when the water temperature exceeds 32°C or drops below 19°C; however, Gulf corals can survive water temperatures exceeding 35–39°C in the summer and falling below 11–9°C in the winter (Coles and Fadlallah, 1991; Spalding et al., 2001; Coles and Riegl, 2012; Riegl and Purkis, 2012). In addition, Gulf corals can survive at high salinity levels, which usually exceed 39 psu in most of the regions of the Arabian Gulf (Coles and Riegl, 2012; Riegl and Purkis, 2012). Very little information is available regarding Gulf coral holobionts, particularly the bacterial communities of these thermally stressed corals (Ashkanani, 2008; Al-Dahash and Mahmoud, 2013).

In our study, we investigated the variations in Actinobacteria associated with the tissue and mucus of various coral hosts thriving under the extreme thermal stress conditions found in the north portion of the Arabian Gulf. The ability of the coral-associated Actinobacteria to produce antimicrobial agents against certain Gram-positive and Gram-negative bacteria was assessed. Furthermore, the temporal and spatial variations in the abundance and diversity of Gulf coral-associated Actinobacteria were investigated.

## Materials and Methods

### Sampling and Sample Processing

The cultured Actinobacteria associated with three different massive coral genera i.e., *Coscinaraea columna, Platygyra daedalea*, and *Porites harrisoni*, were investigated. *C. columna* and *P. daedalea* are listed in the IUCN red list as being of least concern, whereas *P. harrisoni* is listed as being near threatened. All of the species were sampled from the Qit’at Benaya inshore coral reef system north of the Arabian Gulf (N28 37021 E48 25702) in spring (March 2008). The spatial variation in the cultured Actinobacteria associated with the massive brain coral *P. daedalea* was investigated by sampling the tested coral from the Qit’at Benaya inshore reef and the Umm Al-Maradim offshore reef system (N28 40.792 E48 39.105) in autumn (October 2008). In addition, the temporal variation in the cultured Actinobacteria associated with *P. daedalea* was investigated by sampling the tested coral from the inshore reef in March 2008, October 2008, and March 2009. Five colonies of each type of coral were sampled, and three subsamples were collected from each colony. The seawater salinity, pH, temperature, dissolved oxygen, and conductivity were recorded for each site at each sampling day using a Horiba Water Quality Checker (Horiba, USA) (**Supplementary Table S1**).

Samples were collected during spring and autumn during which the corals were not subjected to much stress. It is more likely that the corals sampled at these two seasons would be healthy or at least recovering from the stress during the previous seasons.

Samples of coral tissue and mucus were collected by SCUBA diving. Mucus samples of the corals were collected by sterile syringes, whereas coral nubbins were removed from healthy coral colonies (1 cm^2^ in size patches) and were collected in sterile bags. The coral mucus samples were transferred from the syringes to 15-ml sterile centrifuge tubes, and the volume of the collected mucus was determined. The volume was brought up to 10 ml with phosphate-buffered saline (PBS; Sambrook et al., 1989). In contrast, the coral samples were washed by vigorously shaking the coral tissue with 10 ml of sterile saline water containing 3% NaCl for 2 min to remove the secreted mucus and any attached epiphytes. After washing the samples, the coral nubbin weight was determined, and the coral nubbins (coral tissue + skeleton + mucus) were macerated with a mortar and pestle in 20 ml of sterile PBS, the macerate were referred to through out the study by coral tissue.

### Enumeration of Microbes in the Collected Samples Using the Direct Count Technique

The total numbers of microbes in coral tissue and mucus were determined using the 4’-6-diamidino-2-phenylindole (DAPI) (Sigma, USA) direct count method (Yu et al., 1995; Christensen et al., 1999). An aliquot of 0.25 ml of formaldehyde was added to 5 ml of the seawater samples and to 1 g of the sediment samples, which were suspended in 10 ml of sterile saline water. Additionally, 0.25 ml of formaldehyde was added to 5 ml of the coral tissue suspension and coral mucus samples. The samples were then stained with 0.1 ml of DAPI and incubated in the dark at room temperature for 40 min. Aliquots (50–100 μl) of the stained samples were filtered onto black polycarbonate 0.22-μm membrane filters (Millipore, Ireland) and enumerated by using an epifluorescent microscope (Zeiss, Germany).

### Enumeration of Cultured Actinobacteria in Coral Tissue and Mucus

Serial dilutions of the coral mucus and tissue suspensions were prepared, and the 10^-3^ and 10^-5^ diluents were used. An aliquot of 0.1 ml of each diluent was inoculated on specialized media to promote and maximize the isolation of selected mucus- and coral-associated Actinobacteria. R2A medium (Oxoid, England), M2 medium (Mincer et al., 2002), M4 medium (Zhang et al., 2006), and Starch Casein Agar (SCA) medium (Atlas, 2004) were used, and the R2A and SCA media were modified to contain 3% (w/v) NaCl. The pH of each medium was set to 7.6, and all of the media were supplemented to obtain final concentrations of 50 μg ml^-1^ potassium dichromate (K_2_Cr_2_O_7_), 15 μg ml^-1^ of nalidixic acid, 75 μg ml^-1^ cycloheximide and 75 μg ml^-1^ nystatin. Cycloheximide, potassium dichromate, and nystatin (Sigma, USA) were added to the media to inhibit fungal growth, whereas nalidixic acid was used to inhibit fast-growing Gram-negative bacteria, which would otherwise have overgrown the plates and prevented the isolation of slow-growing Actinobacteria. All of the plates were incubated at 28–30°C for 3–6 weeks. The developed colonies were categorized using morphological and cultural characteristics, counted, and purified.

### Molecular Analysis of the Isolates

The total genomic DNA from the pure bacterial cultures was extracted using the PrepMan Ultra Sample Preparation Reagent (Applied Biosystems, USA) following the manufacturer’s protocol. The DNA extracted from each purified bacterial culture was amplified using PCR techniques. The 16S rRNA gene fragments were amplified using actinobacteria-specific primers. The 16S rRNA genes were amplified using Ready-To-Go PCR Beads (Amersham Biosciences, UK). Each tube contained 25 μl of a reaction mixture composed of 25 ng of the extracted DNA, 25 pmole of each of the forward S-C-Act-235-a-S-20 (CGCGGCCTATCAGCTTGTTG; Stach et al., 2003) and the reverse primers S-C-Act-878-a-A-19 (CCGTACTCCCCAGGCGGGG; Stach et al., 2003) and 23.5 μl of molecular-grade water. PCR amplification was performed in a thermocycler (Applied Biosystems, USA) using PCR programs comprised of an initial denaturation at 95°C for 4 min followed by 30 cycles of 95°C for 30 s, 70°C for 30 s, and 72°C for 30 s and a final extension at 72°C for 7 min (Stach et al., 2003). The amplified PCR product with a size of 643 bp was purified using the QIA Quick Purification Kit (Qiagen, USA) following the manufacturer protocol, and the BigDye Terminator v3.1 Cycle Sequencing Kit (Applied Biosystems, USA) was used for labeling and amplifying the purified product. Two microliters of the sequencing terminator and 2 μl of the 5X Big Dye Sequencing Buffer were mixed with 1 μl of each primer separately and 2 μl of the purified PCR product. The total mixture volume was supplemented with sterile molecular water to reach 10 μl. Using the Big Dye method, the labeling was completed in the GeneAmp PCR system 9700 thermocycler (Applied Biosystems, USA). The PCR program applied included 1 cycle of denaturation at 95°C for 1 min followed by 25 cycles of denaturation at 96°C for 1 min, annealing at 50°C for 5 s and extension at 60°C for 4 min. The labeled products were purified using 3 M sodium acetate (pH 5.2) and absolute ethanol and analyzed using a 3130x*l* genetic analyzer (Applied Biosystems, USA) and the Sequencing Analyzer v5.2 Software (Applied Biosystems, USA). The sequences obtained were compared with other sequences in the GenBank database using BLASTn (Altschul et al., 1997). The sequences were submitted to the GenBank under the accession numbers (KU579016-KU579199).

### Antimicrobial Assays

The agar diffusion test (Isaacson and Kirschbaum, 1986) was used to examine the ability of actinobacterial isolates to produce antimicrobial products. The tests were conducted against indicator strains including Gram-positive (i.e., *Staphylococcus aureus* and *Bacillus* s*ubtilis*) and Gram-negative bacteria (i.e., *Escherichia coli*), which were cultured on marine agar. Two different modifications of the agar diffusion test were applied. The first method included placing disks (i.e., 2 mm in size) of the actinobacterial cultures, with the culture side facing the marine agar, on agar media containing the indicator strains. The second method was the agar-well diffusion test, which depended on making holes in the marine agar that contained the indicator organism and filling the holes with 0.1 ml of 0.45μm filtered marine broth containing the actinobacterial inoculum in the log phase of growth. Positive control (i.e., 100 mg ampicillin, Sigma) and negative control (sterile broth) was also included in the agar-well diffusion test. The plates were incubated at 26°C for 24–48 h, and the actinobacterial activity was evaluated by measuring the inhibition zones on the plates around the disks or the holes.

### Statistical Analysis

Between-sites variations in the actinobacterial abundance was examined using *t-*test and by using SPSS (version 17) software. In addition, within-sites variations and between-hosts variations were examined using *t-*test and one-way ANOVA. Pearson correlation coefficient was used to examine the relationship between the microbial variables in the coral tissue and mucus.

## Results

### Abundance and Diversity of Cultured Actinobacteria Associated with Various Coral Types

**FIGURE 1:**
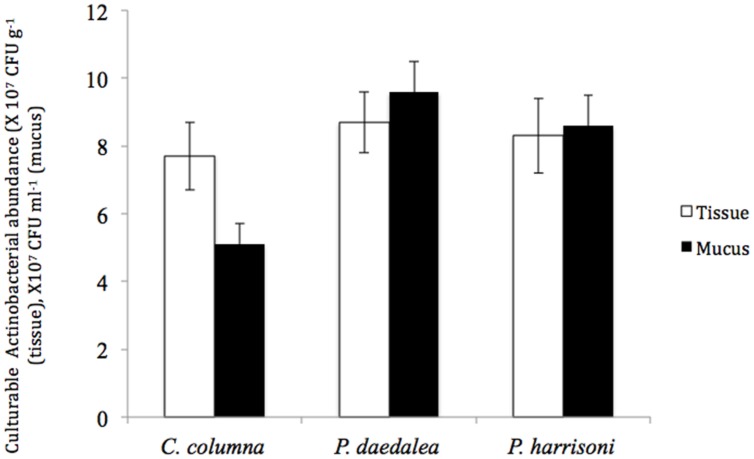
**The abundance of culturable Actinobacteria in coral tissue (□) and mucus (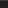) samples collected from the inshore reef system of Qit’at Benaya on March 2008**.

The total numbers of cultured Actinobacteria in *Platygyra daedalea, Porites harrisoni*, and *Coscinaraea columna* in tissue and mucus samples from the inshore reef system coral are shown in **Figure 1.** Different coral hosts were found to harbor equivalent numbers of cultured Actinobacteria in their tissues; in particular, the average numbers detected in tissues of *P. daedalea, P. harrisoni* and *C. columna* were 8.7 × 10^7^ CFU g^-1^, 8.3 × 10^7^ CFU g^-1^, and 7.7 × 10^7^ CFU g^-1^, respectively, and no significant difference was found among the tested corals. Significant differences (*P* < 0.001) in the numbers of cultured Actinobacteria were found in the comparison of mucus samples from various coral hosts; the highest numbers were found in *P. daedalea* mucus samples (9.6 × 10^7^ CFU ml^-1^), and lowest numbers were detected in *C. columna* (5.1 × 10^7^ CFU ml^-1^). In contrast, the comparison of the numbers of cultured Actinobacteria in the coral mucus and tissue samples showed that each coral host harbored significantly different numbers (*P* < 0.01) of Actinobacteria in their tissue and mucus; in particular, higher numbers were found in the coral mucus of both *P. harrisoni* and *P. daedalea* compared with its tissue, whereas *C. columna* harbored significantly less culturable bacteria in its mucus compared with its tissue.

In general, the M4 medium produced the highest numbers and diversity of isolates from the tissue and mucus samples of all of the corals sampled in the current study, whereas the R2A medium yielded the second-highest numbers and diversity, and the SCA medium gave the lowest numbers (**Supplementary Figure S1**).

**FIGURE 2:**
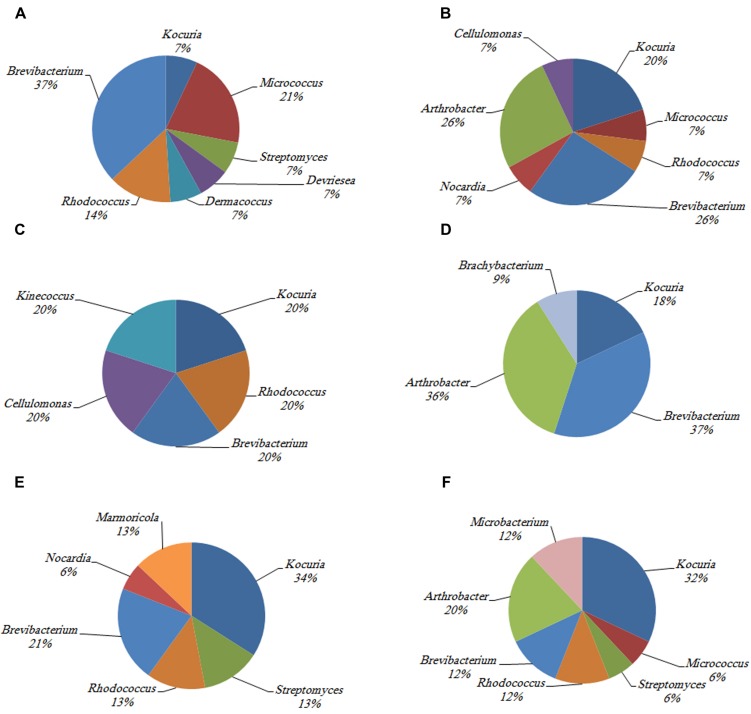
**The identity and percentage of actinobacterial isolates obtained from **(A)***C. culumna* tissue, **(B)***C. culumna* mucus, **(C)***P. deadalea* tissue, **(D)***P. deadalea* mucus, **(E)***P. harrisoni* tissue, and **(F)***P. harrisoni* mucus collected from inshore reef system on March 2008**.

The phylogenetic investigation of 169 isolates obtained from the three investigated hosts showed the dominance of 14 different actinobacterial genera. The similarity between the isolates and their nearest match in GenBank ranged from 97 to 100%. The 14 different genera to which the isolates belong are *Kocuria* sp., *Brevibacterium* sp., *Rhodococcus* sp., *Streptomyces* sp., *Marmoricola* sp., *Nocardia* sp., *Microbacterium* sp., *Arthrobacter* sp., *Micrococcus* sp., *Brachybacterium* sp., *Kineococcus* sp., *Dermacoccus* sp., *Devriesea* sp., and *Cellulomonas* sp. The abundance of different actinobacterial members varied across the samples such that some of these members were significantly more common in particular corals (**Figure 2**).

*Kocuria* sp. and *Brevibacterium* sp. were the most abundant cultured Actinobacteria in the three tested coral hosts. *Dermacoccus* sp. and *Devriesea* sp. were recovered only from the tissue of *C. columna*, whereas *Cellulomonas* sp. was found associated with *C. columna* mucus. *Brachybacterium* sp. and *Kineococcus* sp. were identified in *P. daedalea* mucus and tissue, respectively, whereas *Marmoricola* sp. was detected only in the tissues of both *P. daedalea* and *P. harrisoni*. The results showed that the *P. daedalea* samples harbored less diversity of cultured Actinobacteria than the *C. columna* and *P. harrisoni* samples (**Figure 2**).

### Spatial and Temporal Variation in the Abundance and Diversity of *Platygyra daedalea*-Associated Cultured Actinobacteria

Among the three investigated coral genera, *Platygyra daedalea* showed the highest number but the lowest diversity of culturable Actinobacteria in both the tissue and mucus and was thus selected for further analysis to investigate the spatial and temporal changes in culturable Actinobacteria associated with this type of coral, which is very common in various coral reefs located in the northern section of the Arabian Gulf.

**FIGURE 3:**
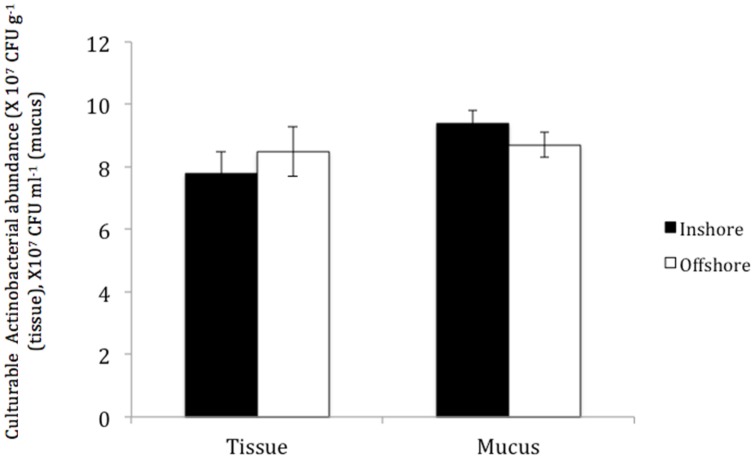
**The abundance of culturable Actinobacteria in coral tissue and mucus samples collected from the inshore reef system of Qit’at Benaya (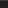) and the offshore reef system of Umm Al-Maradim (□) on October 2008**.

No significant differences were found in the total numbers of actinobacterial isolates obtained from *P. daedalea* tissue and mucus samples obtained from the inshore and offshore reef systems, despite the differences between the two environments. The tissue of *P. daedalea* was found to harbor 7.8 × 10^7^ CFU g^-1^ and 8.5 × 10^7^ CFU g^-1^ in the inshore and offshore reef samples, respectively, whereas the mucus samples obtained from the inshore and offshore reefs harbored 9.4 × 10^7^ CFU ml^-1^ and 8.7 × 10^7^ CFU ml^-1^, respectively (**Figure 3**).

**FIGURE 4:**
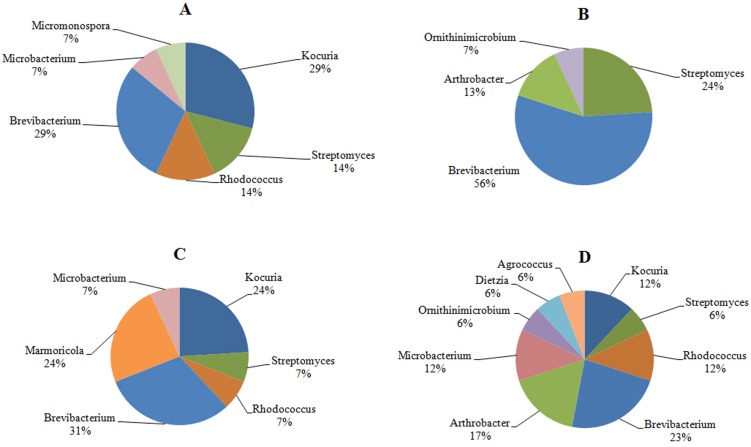
**The identity and percentage of actinobacterial isolates obtained from **(A)***P. deadalea* tissue **(B)***P. deadalea* mucus from inshore reef system of Qit’at Benaya **(C)***P. deadalea* tissue **(D)***P. deadalea* mucus collected from offshore reef system of Umm Al-Maradim (October 2008)**.

The investigation of the phylogenetic diversity of the cultured Actinobacteria associated with the tissue and mucus of *P. daedalea* samples obtained from the inshore (57 isolates) and offshore reef systems (58 isolates) in October 2008 showed a lower diversity in the mucus sample obtained from the inshore reef system (four different genera) compared with that observed in the offshore reef samples (nine different genera; **Figure 4**). However, the tissue samples were found to harbor an equivalent level of diversity (six genera each). The dominance of *Brevibacterium* sp. in the inshore reef and offshore mucus and tissue samples point to the importance of this genus to the coral.

**FIGURE 5:**
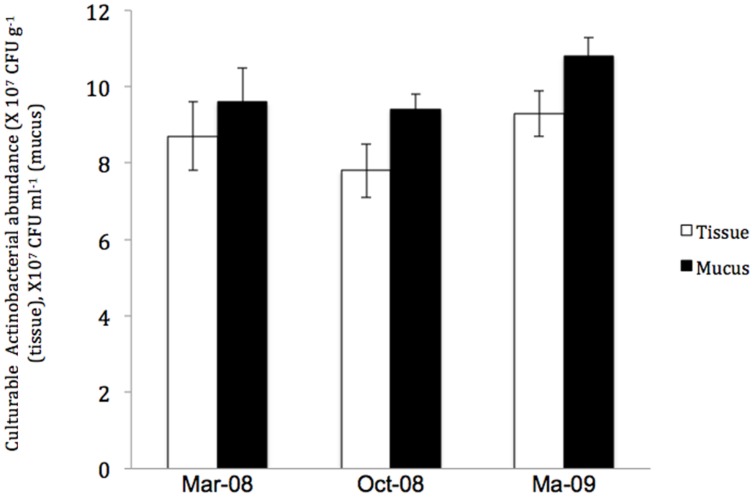
**The abundance of culturable Actinobacteria in *Platygyra daedalea* tissue (□) and mucus (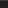) samples collected from the inshore reef system of Qit’at Benaya on March 2009**.

**FIGURE 6:**
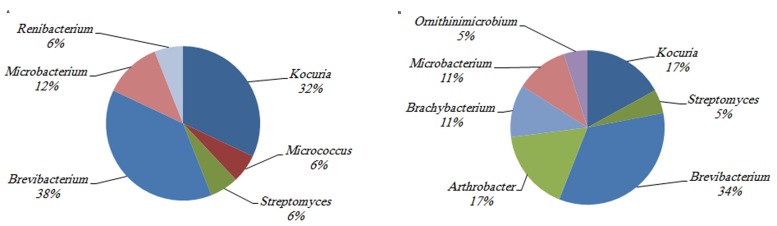
**The identity and percentage of actinobacterial isolates obtained from **(A)***P. deadalea* tissue **(B)***P. deadalea* mucus from Qit’at Benaya inshore reef system (March 2009)**.

In contrast, the investigation of the temporal variation of Actinobacteria in the *P. daedalea* tissue and mucus samples obtained in March 2008, October 2008, and March 2009 showed significant differences in the total numbers of Actinobacteria in the coral tissue and mucus (*P* < 0.01). The highest numbers were recorded in the tissue (9.5 × 10^7^ CFU g^-1^) and mucus (10.8 × 10^7^ CFU ml^-1^) samples obtained in March 2009, whereas the lowest numbers were observed in both the tissue (7.8 × 10^7^ CFU g^-1^) and mucus (9.4 × 10^7^ CFU ml^-1^) samples obtained in October 2008 (**Figure 5**). The variation in the diversity of cultured Actinobacteria among the mucus and tissue samples of *P. daedalea* collected from the inshore reef system at different dates was apparent, as shown in **Figures 2, 4**, and **6**). The tissue samples collected in March 2009 were found to harbor seven different genera, whereas five and six different genera were recorded in the samples collected in March and October 2008, respectively. However, the mucus samples obtained in March 2009 presented the highest diversity with eight different genera, whereas the samples from March and October 2008 showed the presence of only four different genera. Some genera were isolated only once from the tissue samples obtained at the different sampling dates. For example, *Kineococcus* sp. and *Marmoricola* sp. were isolated in March 2008, *Renibacterium* sp. was isolated from the samples collected in March 2009, and *Micromonospora* sp. was isolated from the samples collected in October 2008. Distinctive genera, such as *Brachybacterium* sp. and *Ornithinimicrobium* sp., were found to be associated only with the mucus samples.

### Total Microbial Abundance in Coral Tissue and Mucus

It was important to also quantify the total numbers of microbes in the investigated coral tissue and mucus to estimate the proportion of Actinobacteria in the total microbial community. No significant correlation (*P* > 0.05) was detected between the cultivable Actinobacteria and the total number of microbes in any of the investigated environmental samples. Furthermore, the comparison of the total microbial abundance in the three investigated corals sampled in March 2008 (**Table 1**) showed no significant differences between the total numbers of microbes detected in the tissue and mucus of the three investigated corals. The total numbers of microbes associated with the coral tissue and mucus samples of *P. daedalea* obtained in October 2008 from the inshore and offshore reef systems showed that the microbial numbers in the tested samples obtained from different sites on the same sampling date were significantly different (*P* < 0.001). The highest numbers were recorded in the inshore reef system samples. Significantly different numbers were found in the tissue samples of *P. daedalea* (*P* < 0.001) inhabiting the two sites. Significant differences in the total numbers of microbes were recorded in the tested samples, with the highest and lower numbers being recorded in March 2009 and March 2008, respectively.

**Table 1:** The total number of microbes in coral tissue and mucus, samples from Qit’at Benaya inshore reef and Umm Al-Maradim offshore reef system on various sampling dates.

Site	Date	Coral host	Total count for microbes Mean (min–max) SD	
			
			Coral tissue (×10^9^ cell g^-1^)	Coral mucus (×10^9^ cell ml^-1^)
Inshore reef	March 2008	*Coscinaraea columna*	7.7 (7.0-8.6) 0.7	15.1 (14.3-17.1) 1.2
		*Platygyra daedalea*	5.1 (4.4-6.1) 0.6	12.0 (11.7-12.4) 0.2
		*Porites harrisoni*	4.4 (3.8-4.8) 0.3	15.6 (14.9-16.7) 0.8
	October 2008	*Platygyra daedalea*	7.3 (6.4-9.5) 1.2	14.8 (13.6-15.6) 0.7
	March 2009	*Platygyra daedalea*	8.3 (6.3-9.9) 1.3	17.2(14.4-19.3) 2.4
Offshore reef	October 2008	*Platygyra daedalea*	6.9 (6.4-7.5) 0.4	7.3 (7.1-7.5) 0.1


*Min, minimum; max, maximum; SD, standard deviation.*

### Antimicrobial Activity Potential of Coral-Associated Actinobacteria

**FIGURE 7:**
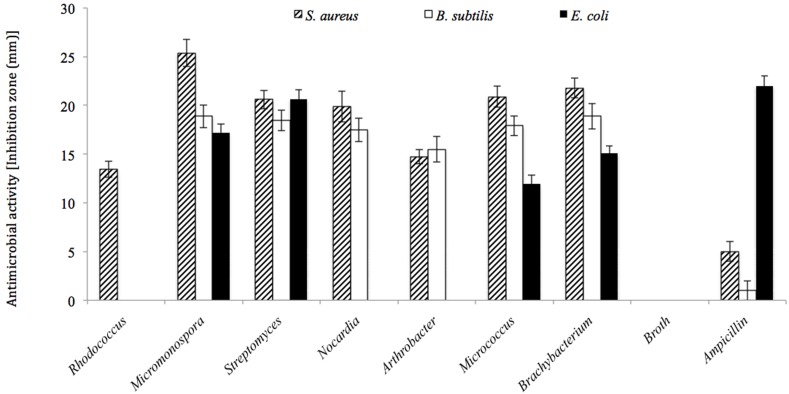
**Antimicrobial activities for the actinobacterial isolates against three tested bacteria (i.e., *Bacillus subtilis*, *Staphylococcus aureus*, and *Escherichia coli*).** All actinobacterial isolates showed strong antimicrobial activities with inhibition zones higher than 15 mm at least to one of the tested bacteria.

**FIGURE 8:**
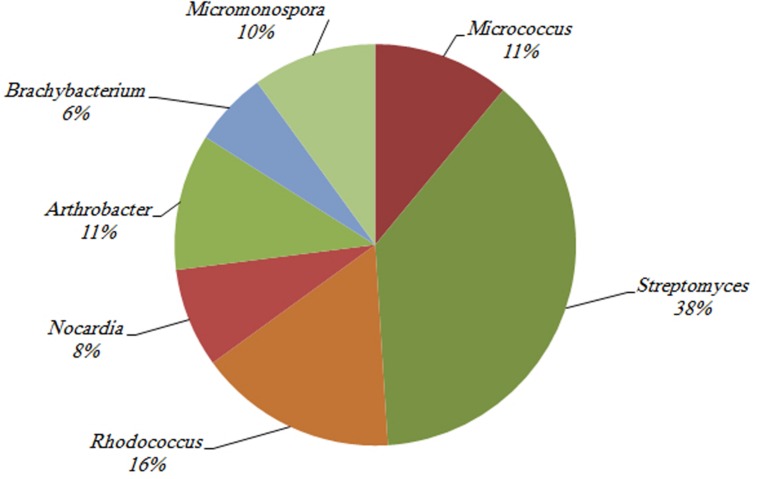
**The percentage of actinobacterial isolates that showed antimicrobial activities against at least one of the utilized tested organisms (i.e., *Bacillus subtilis*, *Staphylococcus aureus*, and *Escherichia coli*)**.

Among the 342 actinobacterial isolates obtained in the study, 82 exhibited antimicrobial activity against at least one tested bacterial culture, i.e., *Staphylococcus aureus, Bacillus subtilis*, or *Escherichia coli* as shown in **Figure 7.** The isolates that were able to produce antimicrobial activities included seven different genera, i.e., Streptomyces (38%), *Rhodococcus* sp. (16%), *Micrococcus* sp. (11%), *Arthrobacter* sp. (11%), *Micromonospora* sp. (10%), *Nocardia* sp. (8%), and *Brachybacterium* sp. (6%) (**Figure 8**). The majority of *Streptomyces, Micrococcus, Micromonospora*, and *Brachybacterium* were able to inhibit the growth of the three tested bacteria, whereas *Arthrobacter* and *Nocardia* were able to inhibit the growth of only the two tested Gram-positive bacteria. In addition, *Rhodococcus* isolates were able to inhibit the growth of *Bacillus subtilis* only. The majority of isolates showed strong antimicrobial activities against the tested organisms where the inhibition zone formed exceeded 15 mm (**Figure 7**). The isolates of each genus showed variations in the level of inhibition against the tested bacteria. For instance, among the 31 tested *Streptomyces* isolates some showed very strong inhibition against *S. aureus*, whereas others could not inhibit the growth of this bacterium (**Supplementary Figure S2**).

## Discussion

The analysis of the abundance and diversity of culturable Actinobacteria associated with *Platygyra daedalea* samples collected between March 2008 and March 2009 from inshore and offshore reef systems located in the north section of the Arabian Gulf revealed higher abundance and diversity of Actinobacteria in the tissue and mucus of this coral more than previously recorded for corals from tropical waters. The results obtained from two other massive Gulf corals, namely *Porites harrisoni* and *Coscinaraea columna*, sampled in March 2008 from the inshore reef system supported this finding. Gulf corals harbor threefold higher numbers of Actinobacteria in their mucus than the amounts that were previously reported by Nithyanand et al. (2011) for corals from the Gulf of Mannar in India. In addition, 82 different isolates belonging to seven different Actinobacterial genera showed antimicrobial activity against at least one Gram-positive or Gram-negative bacterium, and these included some isolates of marine origin that were rarely reported to exhibit antimicrobial activities. These include members of *Rhodococcus*.

Significant differences in the numbers of culturable Actinobacteria were obtained between the mucus and tissue samples of the same coral. Higher numbers were found in the mucus of both *P. daedalea* and *P. harrisoni* compared with the respective tissue samples. This finding opposes that reported by Bourne and Munn (2005), who found similar numbers of culturable bacteria in the coral tissue and mucus. However, the observation from *C. columna* samples, in which higher numbers were detected in the tissue, supports the findings reported by Koren and Rosenberg (2006), who found higher numbers of bacteria in *Oculina patagonica* tissues than in the mucus. Apparently, different coral hosts have their own mechanisms for controlling their symbiont numbers and diversity such that they achieve the maximum benefit from the symbiotic relationship.

*Platygyra daedalea*, *C. columna*, and *P. harrisoni* were found to harbor different numbers of cultivable actinobacteria in their mucus. The highest numbers were recorded in *P. daedalea*, whereas the lowest numbers were found in *C. columna* samples. This difference may be attributed to the amount and rate of mucus secretion by the corals. The rate of mucus production by massive spherical coral species, such as *Platygyra*, is higher than that by hemispherical corals, such as *Porites* (Richman et al., 1975). *Platygyra* contains thicker mucus layers (700-μm thick) than other members of the Faviidae family, which have thinner layers (∼490 μm; Jatkar et al., 2010). The chemical composition of the mucus of the three different coral hosts may be different, thus favoring different microbial populations. This finding is supported by the study conducted by Rohwer et al. (2002), who have found that the mucus of different corals harbors different microbial populations depending on its chemical composition.

Despite harboring lower numbers of cultivable Actinobacteria, the *C. columna* tissue and mucus samples exhibited more Actinobacterial diversity than the *P. daedalea* samples obtained in March 2008. It is worth noticing that there are no contradictions in terms of the high bacterial numbers with low diversity observed in the *P. daedalea* samples. Other researchers have reported similar observations in other aquatic environments and have ascribed this phenomenon to the lack of competition for space and resources, resulting in microbial numbers equivalent to or even higher than those recorded in corresponding environments with higher microbial diversity (Mahmoud et al., 2005). The low Actinobacterial diversity in *Platygyra* samples obtained in March 2008 may suggest that this type of coral is more selective toward its symbionts than *C. columna* and *P. harrisoni*. It may also reflect the variation in the coral immunity levels between the tested corals. *Platygyra* may exhibit a stronger immunity level than the other two corals. Unfortunately, there are no published data to support or refute such an assumption. It is well known that corals are limited to innate immunity, through which they employ physiochemical barriers, such as mucus layers, which act as coral cellular defenses with the ability to distinguish between coral cells and other organism cells in the holobiont and produce both natural and inducible humoral defenses (Sutherland et al., 2004) to protect themselves. Kelman et al. (2006) suggested that scleractinian corals from the Red Sea may rely on non-chemical defenses against microorganisms that may include mucus production and sloughing. Because *Platygyra*, as mentioned previously, produce more and thicker mucus layers than the other corals examined in the current study, this coral may rely widely on this technique to defend itself against pathogens, whereas others that lack this feature depend largely on their symbionts to enhance their immunity.

As mentioned above, different coral hosts harbor similar numbers but present different diversities of cultivable Actinobacteria in their tissues. Ritchie and Lewis (2005) and Guppy and Bythell (2006) have shown that different coral hosts from the same sampling sites may harbor some or no similarities in their bacterial communities. This may also be attributed to coral innate immunity. Although there are no previous reports regarding the coral cellular defenses of the three tested corals, it is possible that the corals investigated in the current study allow selected symbionts to reach certain numbers in their tissue, where they keep these numbers under control and any excess can either be digested during feeding or repelled into the mucus. This is in agreement with the scenario suggested by Baghdasarian and Muscatine (2000), who have reported that healthy cnidarians expel actively dividing zooxanthellae cells into the mucus to maintain a constant algal population density within the host tissue. However, the variation in the coral-associated actinobacterial diversity can be attributed to the individuality of each host.

*Brevibacterium* and *Kocuria* were the most dominant actinobacterial isolates in the investigated coral tissue and mucus samples. Phylogenetic trees constructed from Gulf-coral *Brevibacterium* and *Kocuria* and their counterparts from other environments revealed that the Gulf isolates are unique. The Brevibacterium phylogenetic tree (**Supplementary data Figure S3**) showed more than 70 Gulf coral-associated isolates clustering together and far from Brevibacterium from other environments. One exception when *B. mcbrellneri* (NZ-ADNU010000), an isolate from human urogenital tract, is considered. Kocuria on the other hand, showed variation among Gulf isolates but, all Gulf isolates clustered separately from their counterparts from other environments except for the airborne isolates *K. turfanensis* (DQ531634) and *K. flava* (EF602041) (**Supplementary data Figure S4**). Some studies have found an association between *Brevibacterium* and coral samples (Sabdono and Radjasa, 2008; Seemann et al., 2009). *Kocuria* has also been isolated from coral mucus (Ritchie, 2006) and tissue (Sabdono et al., 2005). The reason underlying why these two genera were found to dominate the cultivable actinobacterial groups is unknown. Mimura and Nagata (2001) have reported that *Brevibacterium* sp. JCM 6894 from seawater can more efficiently degrade the water-soluble fraction of jellyfish than other bacteria. These bacteria also degrade organophosphorus pesticides (Sabdono and Radjasa, 2008). In view of these abilities, *Brevibacterium* was suggested by Mimura and Nagata (2001) to be a strong candidate for use in bioremediation strategies. Could it be possible that the capability of *Brevibacterium* to degrade various chemical compounds facilitates their successful association with corals? Recent studies have shown that coral-associated *Brevibacterium* (Seemann et al., 2009) are able to produce palytoxin (PTX) such that it can accumulate in the tissue of the marine animals that feed on corals (Gleibs and Mebs, 1999; Seemann et al., 2009). Is it possible that corals accommodate these toxin producers to participate in reducing the grazing pressure exerted by other marine animals on corals? Or it is only a coincidence that the most dominant Actinobacteria are associated with corals that produce PTX? The literature has not revealed any special role of *Kocuria* in the marine system. *Kocuria* has been described as a marine organism (Kim et al., 2004), but only a few papers have reported its occurrence in the marine environment, and even fewer papers have reported its association with corals.

Although no significant differences were found in the number of culturable Actinobacteria between the inshore and offshore reef systems, a higher diversity was found in the offshore *P. daedalea* mucus samples collected in October 2008. Coral-associated microbial communities present differences with changing depth, water quality, and geographic location (Rohwer et al., 2001, 2002; Frias-Lopez et al., 2002; Reshef et al., 2006; Klaus et al., 2007). Therefore, variations would be expected in the actinobacterial diversity associated with the same coral host occupying different sites. In addition, changes in coral genotypes between the two sites may provide an explanation for the variation in their associated microbes, including Actinobacteria. This phenomenon of genotype variation is supported by the DGGE findings reported by Rohwer et al. (2001), who have shown that the microbial populations of 25 *Montastraea franksi* colonies from five different reef systems share only one common band due to variations in the coral genotypes. However, the species-specific microbiota principle suggested by Ritchie and Smith (1997) and Rohwer et al. (2001, 2002) should not be neglected. The results of the current study showed that the same coral samples of different individuals collected from two sites shared a number of identical actinobacterial genera, and this number was higher than that detected in both mucus and tissue samples of the same individual.

The total numbers of microbes in various environmental samples were higher in the inshore reef than the offshore reef system. This finding may be attributed to the high sewage input seeding the inshore water with high numbers of microbes, which may have an indirect effect on coral health in the area. It has been documented that the inshore reefs of Kuwait are less healthy than their offshore counterparts (Carpenter et al., 1997; Ashkanani, 2008; Al-Sarraf, 2009). Unfortunately, the correlation test did not reveal any significant correlation between the total numbers of microbes and the numbers of culturable Actinobacteria in the coral samples. Therefore, no direct relationship can be established between the two variables.

In contrast, the temporal investigation of *P. daedalea-*associated culturable Actinobacteria showed higher diversity and numbers of culturable Actinobacteria and total numbers of microbes in the mucus and tissue samples collected in March 2009, whereas the lowest numbers were recorded in the samples collected in November 2008. A natural variation in coral communities is expected to be observed over time, and many studies that monitored certain reef systems for a sufficiently long time have reported that disturbing these systems due to various man-made or natural factors results in alterations in coral abundance and survival (Connell et al., 1997). However, few studies have attempted to explain how this alteration affects the microbial population of the corals themselves. The increment in water temperature to levels exceeding certain thresholds leads to significant changes in the mucus bacterial population (Ritchie and Smith, 1995; Guppy and Bythell, 2006) due either to decomposition of the coral mucus with extracellular proteases (Bourne and Munn, 2005) or to a reduction in the antibiotic content of the coral mucus (Ritchie, 2006). The “Coral Probiotic Hypothesis” suggested by Reshef et al. (2006) may provide an explanation for the changes in the actinobacterial abundance and diversity of *P. daedalea* sampled from the inshore reef system at different times. Under this hypothesis, corals experiencing changes in environmental conditions adapt rapidly by changing their microbial partners to accommodate more antimicrobial producers. By doing so, corals gain the ability to develop resistance to pathogens.

Nithyanand and Pandian (2009) reported that actinomycetes associated with corals and their produced metabolites had not yet been explored, and since then, few studies have focused on this topic, but all of these targeted corals from tropical regions. Our study targeted the actinobacterial community of the thermally stressed corals of the Arabian Gulf. The results showed that *Streptomyces*-related isolates dominated (∼38%) the group of isolates with antimicrobial activities, even though *Streptomyces* were not the cultured Actinobacteria that dominated the tissue and mucus of Gulf corals. This is expected because more than 500 species of *Streptomyces* account for 70–80% of secondary metabolites and it is well documented that marine *Streptomyces* are able to produce bioactive compounds with a range of activities, including anticancer, antimicrobial, and enzyme inhibition functions (Lam, 2006; Solanki et al., 2008). The second most dominant genus in this group was *Rhodococcus*, which made up 16% of the total isolates with antimicrobial activities. This is an interesting finding because papers reporting the ability of isolates of this genus from marine origin to produce antimicrobial products are few (Zhang et al., 2013). In addition to *Rhodococcus*, few have reported the antimicrobial activity potential of *Brachybacterium* (Radjasa, 2007). In the current study, 6% of the isolates with antimicrobial activities were related to *Brachybacterium*. Radjasa (2007) has investigated sponge-associated Actinobacteria that had 99% 16S rRNA-gene similarity to *Brachybacterium rhamnosum* and reported their ability to contain polyketide synthase (PKS) and non-ribosomal peptide synthase (NRPS) responsible for structurally synthesizing bioactive secondary metabolites and to inhibit the growth of *E. coli*. It is likely that novel isolates and new findings will be obtained because the isolates in this study showed antimicrobial activities against *E. coli, S. aureus*, and *B. subtilis* and were related to *B. paraconglomeratum*, *B. phenoliresistens*, and *B. zhongshanense*. In other words, they are quite different from that reported by Radjasa (2007). *Arthrobacter*-related isolates, which made up 11% of the total isolates with antimicrobial activities, deserve attention. The ability of *Arthrobacter* to produce antibiotics has been reported previously by a few investigators working on isolates of marine origin. However, Shnit-Orland and Kushmaro (2008) reported that *Micrococcus* and *Arthrobacter* isolated from corals showed no antimicrobial activities. Hentschel et al. (2001) obtained an isolate from a Mediterranean sponge, whereas Radjasa et al. (2008) isolated an *Arthrobacter* species from corals of the North Java Sea that shows antimicrobial activities. Even though *Rhodococcus* and *Arthrobacter* are common soil Actinobacteria, their marine counterparts appear to have more antimicrobial potential than the terrestrial ones, which agrees with the conclusions reported by Lam (2006).

The other three actinobacterial genera that showed antimicrobial activities, namely *Micromonospora*, *Micrococcus*, and *Nocardia*, were previously isolated from various marine habitats and were reported to be a potential source of bioactive compounds (Bultel-Poncé et al., 1998; Hentschel et al., 2001; Lam, 2006, 2007; Radjasa et al., 2008; Solanki et al., 2008; Nithyanand and Pandian, 2009; Olano et al., 2009). It is likely that some of the isolates obtained in the current study contain novel compounds that have not previously been described. Even though *Brevibacterium* and *Kocuria* were the most dominant actinobacterial isolates, they failed to show any antimicrobial activity, whereas less dominant genera, such as *Streptomyces*, had antimicrobial activity.

## Conclusion

The variations in the culturable actinobacterial populations associated with corals in inshore and offshore reef systems of the north section of the Arabian Gulf were observed. Different coral host types harbored different cultivable actinobacterial populations. Differences in the abundance and diversity of Actinobacteria were detected between the mucus and tissue of the same coral host. In addition, temporal and spatial variations in the abundance and diversity of the cultivable actinobacterial population were detected. Focusing on the diversity of coral-associated Actinobacteria may lead to the discovery of novel antimicrobial metabolites with potential biotechnological applications.

## Author Contributions

All authors listed, have made substantial, direct and intellectual contribution to the work, and approved it for publication.

## Conflict of Interest Statement

The authors declare that the research was conducted in the absence of any commercial or financial relationships that could be construed as a potential conflict of interest.

## References

[B1] Al-Dahash L. M. Mahmoud H. M. (2013). Harboring oil-degrading bacteria: a potential mechanism of adaptation and survival in corals inhabiting oil-contaminated reefs. *Mar. Pollut. Bull.* 72 364–374. 10.1016/j.marpolbul.2012.08.029 23014479

[B2] Al-Sarraf M. J. (2009). *Molecular Phylogeny and Community Fingerprinting of Systematic Zooxanthellae of the Kuwaiti Coral Reefs.* M.Sc. thesis, Kuwait University, Kuwait City.

[B3] Altschul S. F. Madden T. L. Schäfer A. A. Zhang J. Zhang Z. Miller W. (1997). Gapped BLAST and PSL-BLAST: a new generation of protein database search programs. *Nucleic Acids Res.* 25 3389–3402. 10.1093/nar/25.17.3389 9254694 PMC146917

[B4] Ashkanani A. M. G. (2008). *Studying the Diversity of Bacterial Communities Associated with Coral Mucus of Various Coral Genera from the Kuwaiti Water.* M.Sc. thesis, Kuwait University, Kuwait City.

[B5] Atlas M. R. (2004). *Handbook of Microbiological Media.* Washington: CRC Press.

[B6] Baghdasarian G. Muscatine L. (2000). Preferential expulsion of diving algal cells as a mechanism for regulation algal-cnidarian symbiosis. *Biol. Bull.* 199 278–286. 10.2307/1543184 11147708

[B7] Banin E. Israely T. Fine M. Loya Y. Rosenberg E. (2001). Role of endosymbiotic zooxanthellae and coral mucus in the adhesion of the coral-bleaching pathogen *Vibrio shiloi* to its host. *FEMS Microbiol. Lett.* 199 33–37. 10.1111/j.1574-6968.2001.tb10647.x 11356564

[B8] Blunt J. W. Copp B. R. Hu W. P. Munro M. H. Northcote P. T. Prinsep M. R. (2007). Marine natural products. *Nat. Prod. Res.* 24 31–86. 10.1039/b603047p 17268607

[B9] Blunt J. W. Copp B. R. Munro M. H. Northcote P. T. Prinsep M. R. (2006). Marine natural products. *Nat. Prod. Res.* 23 26–78. 10.1039/b502792f 16453031

[B10] Bourne D. G. Munn C. B. (2005). Diversity of bacteria associated with the coral *Pocillopora damicornis* from the Great Barrier Reef. *Environ. Microbiol.* 7 1162–1174. 10.1111/j.1462-2920.2005.00793.x 16011753

[B11] Bull A. T. Stach J. E. Ward A. C. Goodfellow M. (2000). Search and discovery strategies for biotechnology: the paradigm shift. *Microbiol. Mol. Biol. Rev.* 64 573–606. 10.1128/MMBR.64.3.573-606.2000 10974127 PMC99005

[B12] Bultel-Poncé V. Debitus C. Berge J. Cerceau C. Guyot M. (1998). Metabolites from the sponge-associated bacterium *Micrococcus luteus*. *J. Mar. Biotechnol.* 6 233–236. 9852617

[B13] Carpenter K. E. Harrison P. L. Hodgson G. Alsaffar A. H. Alhazeem S. H. (1997). *The Corals and Coral Reef Fishes of Kuwait.* Kuwait City: Kuwait Institute for Scientific Research.

[B14] Caundliffe E. (2006). Antibiotic production by actinomycetes: the *Janus* faces of regulation. *J. Ind. Microbiol. Biotechnol.* 33 500–506. 10.1007/s10295-006-0083-6 16463161

[B15] Christensen H. Hansen M. Sorensen J. (1999). Counting and size classification of active soil bacteria by fluorescence in situ hybridization with an rRNA oligonucleotide probe. *Appl. Environ. Microbiol.* 65 1753–1761. 10103277 10.1128/aem.65.4.1753-1761.1999 PMC91247

[B16] Coles S. L. Fadlallah Y. H. (1991). Reef coral survival and mortality at low temperatures in the Arabian Gulf: new species-specific lower temperature limits. *Coral Reefs* 9 231–237. 10.1007/BF00290427

[B17] Coles S. L. Riegl B. M. (2012). Thermal tolerances of reef corals in the Gulf: a review of the potential for increasing coral survival and adaptation to climate change through assisted translocation. *Mar. Pollut. Bull.* 72 323–332. 10.1016/j.marpolbul.2012.09.006 23058810

[B18] Connell J. H. Hughes T. P. Wallace C. C. (1997). A 30-year study of coral abundance, recruitment, and disturbance at several scales in space and time. *Ecol. Monogr.* 67 461–488. 10.1890/0012-9615(1997)067[0461:AYSOCA]2.0.CO;2

[B19] de Castro A. P. Araújo S. D. Jr. Reis A. M. Moura R. L. Francini-Filho R. B. Pappas G. Jr. (2010). Bacterial community associated with healthy and diseased reef coral *Mussismilia hispida* from eastern Brazil. *Microb. Ecol.* 59 658–667. 10.1007/s00248-010-9646-1 20352207

[B20] Ducklow H. W. Mitchell R. (1979). Bacterial populations and adaptions in the mucus layers of living corals. *Limnol. Oceanogr.* 24 715–725. 10.4319/lo.1979.24.4.0715

[B21] Frias-Lopez J. Zerkle A. L. Bonheyo G. T. Fouke B. W. (2002). Partitioning of bacterial communities between seawater and healthy, black band diseased, and dead coral surfaces. *Appl. Environ. Microbiol.* 68 2214–2228. 10.1128/AEM.68.5.2214-2228.2002 11976091 PMC127591

[B22] Gleibs S. Mebs D. (1999). Distribution and sequestration of palytoxin in coral reef animals. *Toxicon* 37 1521–1527. 10.1016/S0041-0101(99)00093-8 10482387

[B23] Guppy R. Bythell L. C. (2006). Environmental effects on bacterial diversity in the surface mucus layer of the reef coral *Montastraea faveolata*. *Mar. Ecol. Prog. Ser.* 328 133–142. 10.3354/meps328133

[B24] Hentschel U. Schmid M. Wagner M. Fieseler L. Gernert C. Hacker J. (2001). Isolation and phylogenetic analysis of bacteria with antimicrobial activities from the Mediterranean sponges *Aplysina aerophoba* and *Aplysina cavernicola*. *FEMS Microbiol. Ecol.* 35 305–312. 10.1111/j.1574-6941.2001.tb00816.x 11311441

[B25] Isaacson D. M. Kirschbaum J. (1986). “Assays of antimicrobial substances,” in *Manual of Industrial Microbiology and Biotechnology*, eds Demain A. L. Solomon N. A. (Washington, DC: ASM), 410–435.

[B26] Jatkar A. A. Brown B. E. Bythell J. C. Guppy R. Morris N. J. Pearson J. P. (2010). Measuring mucus thickness in reef corals using a technique devised for vertebrate applications. *Mar. Biol.* 157 261–267. 10.1007/s00227-009-1313-z

[B27] Kelman D. Kashman Y. Rosenberg E. Kushmaro A. Yossi Loya Y. (2006). Antimicrobial activity of Red Sea corals. *Mar. Biol.* 149 357–363. 10.1007/s00227-005-0218-8

[B28] Kim S. B. Nedashkovskaya O. Mikhailov V. V. Han S. K. Kim K. Rhee M. (2004). *Kocuria marina* sp. nov., a novel actinobacterium isolated from marine sediment. *Int. J. Syst. Evol. Microbiol.* 54 1617–1620. 10.1099/ijs.0.02742-0 15388718

[B29] Kinsman D. J. J. (1964). Reef coral tolerance of high temperatures and salinities. *Nature* 202 1280–1282. 10.1038/2021280a0

[B30] Klaus J. S. Janse I. Heikoop J. M. Sanford R. A. Fouke B. W. (2007). Coral microbial communities, zooxanthellae and mucus along gradients of seawater depth and coastal pollution. *Environ. Microbiol.* 9 1291–1305. 10.1111/j.1462-2920.2007.01249.x 17472641

[B31] Kooperman N. Ben-Dov E. Kramarsky-Winter E. Barak Z. Kushmaro A. (2007). Coral mucus-associated bacterial communities from natural and aquarium environments. *FEMS Microbiol. Lett.* 276 106–113. 10.1111/j.1574-6968.2007.00921.x 17937669

[B32] Koren O. Rosenberg E. (2006). Bacteria associated with mucus and tissues of the coral *Oculina patagonica* in summer and winter. *Appl. Environ. Microbiol.* 72 5254–5259. 10.1128/AEM.00554-06 16885273 PMC1538755

[B33] Kushmaro A. Loya Y. Fine M. Rosenberg E. (1996). Bacterial infection and coral bleaching. *Nature* 380 396 10.1038/380396a0

[B34] Lam K. S. (2006). Discovery of novel metabolites from marine actinomycetes. *Curr. Opin. Microbiol.* 9 245–251. 10.1016/j.mib.2006.03.004 16675289

[B35] Lam K. S. (2007). New aspects of natural products in drug discovery. *Trends Microbiol.* 15 279–289. 10.1016/j.tim.2007.04.001 17433686

[B36] Lampert Y. Kelman D. Dubinsky Z. Nitzan Y. Hill R. T. (2006). Diversity of culturable bacteria in the mucus of the Red Sea coral Fungia scutaria. *FEMS Microbiol. Rev.* 58 99–108. 10.1111/j.1574-6941.2006.00136.x 16958911

[B37] Mahmoud H. M. A. Goulder R. Carvalho G. R. (2005). The response of epilithic bacteria to different metal regime in two upland streams: assessed by conventional microbiological methods and PCR-DGGE. *Arch. Hydrobiol.* 163 405–427. 10.1127/0003-9136/2005/0163-0405

[B38] Manivasagan P. Kang K.-H. Sivakumar K. Li-Chan E. C.-Y. Oh H.-M. Kim S.-K. (2014). Marine actinobacteria: an important source of bioactive natural products. *Environ. Toxicol. Pharmacol.* 38 172–188. 10.1016/j.etap.2014.05.014 24959957

[B39] Manivasagan P. Venkatesan J. Sivakumar K. Kim S.-K. (2013). Marine actinobacterial metabolites: current status and future perspectives. *Microbiol. Res.* 168 311–332. 10.1016/j.micres.2013.02.002 23480961

[B40] Mimura H. Nagata S. (2001). Degradation of water-soluble fraction of jellyfish by a marine bacterium, *Brevibacterium* sp. JCM 6894. *Microb. Environ.* 16 121–123. 10.1264/jsme2.2001.121

[B41] Mincer T. J. Jennsen P. R. Kauffman C. A. Fenical W. (2002). Widespread and persistent populations of a major new marine actionbacteria taxon in ocean sediments. *Appl. Environ. Microbiol.* 68 5005–5011. 10.1128/AEM.68.10.5005-5011.2002 12324350 PMC126404

[B42] Nithyanand P. Manju S. Pandian S. K. (2011). Phylogenetic characterization of culturable actinomycetes associated with the mucus of the coral *Acropora digitifera* from Gulf of Mannar. *FEMS Microbiol. Lett.* 314 112–118. 10.1111/j.1574-6968.2010.02149.x 21105906

[B43] Nithyanand P. Pandian S. K. (2009). Phylogenetic characterization of culturable bacterial diversity associated with the mucus and tissue of the coral *Acropora digitifera* from the Gulf of Mannar. *FEMS Microbiol. Ecol.* 69 384–394. 10.1111/j.1574-6941.2009.00723.x 19619231

[B80] Nithyanand P. Thenmozhi R. Rathna J. Pandian S. K. (2010). Inhibition of *Streptococcus pyogenes* biofilm formation by coral-associated actinomycetes. *Curr. Microbiol.* 60 454–460. 10.1007/s00284-009-9564-y 20020301

[B44] Olano C. Méndez C. Salas J. A. (2009). Antinumor compounds from marine actinomycetes. *Mar. Drugs* 11 210–248. 10.3390/md7020210 19597582 PMC2707044

[B45] Paul J. H. Deflaun M. F. Jeffery W. H. (1986). Elevated levels of microbial activity in the coral surface monolayer. *Mar. Ecol. Prog. Ser.* 33 29–40. 10.3354/meps033029

[B46] Penn K. Wu D. Eisen J. A. Ward N. (2006). Characterization of bacterial communities associated with Deep-Sea on Gulf of Alaska Seamounts. *Appl. Environ. Microbiol.* 72 1680–1683. 10.1128/AEM.72.2.1680-1683.2006 16461727 PMC1392894

[B47] Piskorska M. Smith G. Weil E. (2007). Bacteria associated with the coral *Echinopora lamellosa* (Esper 1795) in the Indian Ocean Zanzibar Region. *Afr. J. Environ. Sci. Technol.* 1 93–98.

[B48] Piza F. F. Prado P. I. Manfio G. P. (2004). Investigation of bacterial diversity in Brazilian tropical estuarine sediments reveals high actinobacterial diversity. *Antonie Van Leeuwenhoek* 86 317–328. 10.1007/s10482-005-0162-0 15702384

[B49] Radjasa O. K. (2007). Antimicrobial activity of sponge associated-bacteria isolated from north Java Sea. *J. Coast. Dev.* 10 143–150.

[B50] Radjasa O. K. Wiese J. Sabdono A. Imhoff J. F. (2008). Corals as source of bacterial with antimicrobial activity. *J. Coast. Dev.* 11 121–130.

[B51] Reshef L. Koren O. Loya Y. Zilber-Rosenberg I. Rosenberg E. (2006). The coral probiotic hypothesis. *Environ. Microbiol.* 8 2068–2073. 10.1111/j.1462-2920.2006.01148.x 17107548

[B52] Richman S. Loya Y. Slobodkin L. B. (1975). The rate of mucus production by corals and its assimilation by the coral reef copepod *Acartia negligens*. *Limnol. Oceanogr.* 20 918–923. 10.4319/lo.1975.20.6.0918

[B53] Riegl B. M. Purkis S. J. (2012). *Coral Reefs of the Gulf: Adaptation to Climatic Extremes.* Dordrecht: Springer.

[B54] Ritchie A. E. (2006). Regulation of microbial populations by coral surface mucus and mucus-associated bacteria. *Mar. Ecol. Prog. Ser.* 322 1–14. 10.3354/meps322001

[B55] Ritchie A. E. Lewis T. (2005). Bacterial composition of surface associated microbes found in three Hawaiian coral species: *Porites compressa*, *Porites lobata*, and *Montipora capitata*. *J. Young Investig.* 12 4.

[B56] Ritchie K. B. Smith G. W. (1995). Preferential carbon utilization by surface bacterial communities from water mass, normal, and white-band diseased *Acropora cervicornis*. *Mol. Mar. Biol. Biotechnol.* 4 345–354.

[B57] Ritchie K. B. Smith G. W. (1997). “Physiological comparison of Bacterial communities from various species of scleractinian corals,” in *Proceeding from 8th International Coral Reef Symposium* Vol. 1 eds Lessios H. A. Macintyre I. G. (Panama: Smithsonian Tropical Research Institute), 521–526.

[B58] Ritchie K. B. Smith G. W. (2004). “Microbial communities of coral surface mucopolysaccharide layers,” in *Coral Health and Disease*, eds Rosenberg E. Loya Y. (New York, NY: Springer-Verlag), 259–263.

[B59] Rohwer F. Breitbart M. Jara J. Azam F. Knowlton N. (2001). Diversity of bacterial associated with the Caribbean coral *Montastraea franksi*. *Coral Reefs* 20 85–91. 10.1007/s003380100138

[B60] Rohwer F. Seguritan V. Azam F. Knowlton N. (2002). Diversity and distribution of coral-associated bacteria. *Mar. Ecol. Prog. Ser.* 243 1–10. 10.3354/meps243001

[B61] Sabdono A. Radjasa O. K. (2008). Phylogenetic diversity of organophosphorous pesticide-degrading coral bacteria from mid-west coast of Indonesia. *Biotechnology* 7 694–701. 10.3923/biotech.2008.694.701

[B62] Sabdono A. Radjasa O. K. Stöhr R. Zocchi E. (2005). Diversity of culturable bacterial community associated with the coral *Galaxea fascicularis* from Ujung Lulon, Indonesia. *J. Coast. Dev.* 9 57–63.

[B63] Sambrook J. Fritsch E. F. Maniatis T. (1989). *Molecular Cloning: A Laboratory Manual*, 2nd Edn New York, NY: Cold Spring Harbor Laboratory Press.

[B64] Seemann P. Gernert C. Schmitt S. Mebs D. Hentschel U. (2009). Detection of hemolytic bacteria from *Palythoa caribaeorum* (Cnidaria, Zoantharia) using a novel palytoxin-screening assay. *Antonie Van Leeuwenhoek* 96 405–411. 10.1007/s10482-009-9353-4 19504172

[B65] Shashar N. Cohen Y. Loya Y. Sar N. (1994). Nitrogen fixation (acetylene reduction) in stony corals: evidence for coral–bacterial interactions. *Mar. Ecol. Prog. Ser.* 111 259–264. 10.3354/meps111259

[B66] Sheppard C. Price A. Roberts C. (1992). *Marine Ecology of the Arabian Region: Patterns and Processes in Extreme Tropical Environments.* London: Academic Press.

[B67] Shnit-Orland M. Kushmaro A. (2008). “Coral mucus bacteria as a source for antibacterial activity,” in *Proceedings of the 11th International Coral Reef Symposium* Vol. 1 Ft. Lauderdale, FL, 257–259.

[B68] Sivakumar K. Sahu M. K. Thangaradjou T. Kannan L. (2007). Research on marine actinobacteria in India. *Ind. J. Microbiol.* 47 186–196. 10.1007/s12088-007-0039-1 PMC3450339 23100666

[B69] Solanki R. Khanna M. Lal R. (2008). Bioactive compounds from marine actinomycetes. *Ind. J. Microbiol.* 48 410–431. 10.1007/s12088-008-0052-z PMC3476783 23100742

[B70] Spalding M. D. Ravilious C. Green E. P. (2001). *World Atlas of Coral Reefs.* Berkeley, CA: University of California Press.

[B71] Stach J. E. Maldonado L. A. Ward A. C. Goodfellow M. Bull A. T. (2003). New primers for the class Actinobacteria: application to marine and terrestrial environments. *Environ. Microbiol.* 5 828–841. 10.1046/j.1462-2920.2003.00483.x 14510836

[B72] Sutherland K. P. Porter J. P. Torres C. (2004). Disease and immunity in Caribbean and Indo-Pacific zooxanthellate corals. *Mar. Ecol. Prog. Ser.* 266 273–302. 10.3354/meps266273

[B73] Webster N. S. Negri D. G. Munro M. M. Battershill C. N. (2004). Diverse microbial community inhabit Antarctic sponges. *Environ. Microbiol.* 6 288–300. 10.1111/j.1462-2920.2004.00570.x 14871212

[B74] Wegley L. Edwards R. Rodriguez-Brito B. Liu H. Rohwer F. (2007). Metagenomic analysis of the microbial community associated with the coral *Porites astreoides*. *Environ. Microbiol.* 9 2707–2719. 10.1111/j.1462-2920.2007.01383.x 17922755

[B75] Williams W. M. Viner A. B. Broughton W. L. (1987). Nitrogen fixation (acetylene reduction) associated with the living coral *Acropora variablis*. *Mar. Biol.* 94 521–535. 10.1007/BF00431399

[B76] Yakimov M. M. Cappello S. Crisafi E. Tursi A. Savini A. Cesare C. (2006). Phylogenetic survey of metabolically active communities associated with the Deep-Sea coral *Lophelia pertusa* from the Apulian plateau, Central Mediterranean Sea. *Deep Sea Res.* 53 62–75. 10.1016/j.dsr.2005.07.005

[B77] Yu W. Dodds W. K. Banks K. Skalsky J. Staruss E. (1995). Optimal and sample storage time for direct microscopic enumeration of total and active bacteria in soil with two fluorescent dyes. *Appl. Environ. Microbiol.* 61 3367–3372. 16535124 10.1128/aem.61.9.3367-3372.1995 PMC1388578

[B78] Zhang H. Lee Y. K. Zhang W. Lee H. K. (2006). Culturable actinobacteria from the marine sponge *Hymeniacidon perleve*: isolation and phylogenetic diversity by 16S rRNA gene-RFLP analysis. *Antonie Van Leeuwenhoek* 90 159–169. 10.1007/s10482-006-9070-1 16871424

[B79] Zhang X.-Y. He F. Wang G.-H. Bao L. Xu X.-Y. Qi S.-H. (2013). Diversity and antibacterial activity of culturable Actinobacteria isolated from five species of the South China Sea gorgonian corals. *World J. Microbiol. Biotechnol.* 29 1107–1116. 10.1007/s11274-013-1279-3 23386321

