# Supplementary material for: Coral-Associated Actinobacteria: Diversity, Abundance, and Biotechnological Potentials
Source: Front Microbiol. 2016 Feb 29;7:204. doi: 10.3389/fmicb.2016.00204 (PMC4770044; doi:10.3389/fmicb.2016.00204)
Supplement: Supplementary file 1 [file Data_Sheet_1.DOCX]

**Supplementary data:**

The seawater quality variables at the day of sampling from both inshore and offshore reef systems are summarized in Table S1.

Table S1. Water quality variables from inshore reef system of Qit’at Benaya sampled March 2008, October 2008 and March 2009 and water quality variables from offshore reef system of Umm Al-Maradim sampled on October 2008.

| **Water quality parameters** | **Inshore March 2008** | **Inshore October 2008** | **Inshore March 2009** | **Offshore October 2008** |
| --- | --- | --- | --- | --- |
| Turbidity (NTU) | 9 | 7 | 5 | 4 |
| Dissolved oxygen (mg L^-1^) | 3.64 | 3.5 | 4.12 | 5.78 |
| pH | 8 | 8.06 | 8.1 | 8.06 |
| Conductivity (ms cm ^-1^) | 54.6 | 57 | 56.8 | 55.7 |
| Salinity (%) | 3.58 | 3.79 | 3.76 | 3.69 |
| Temperature (°C) | 21.2 | 23 | 20.9 | 24.1 |

**Number of isolates identified**

**Number of isolates identified**

Figure S1. The cultivable actinobacterial diversity obtained from different media **A**: *C. culumna* tissue, **B**: *C. culumna* mucus, **C**: *P. harrisoni* tissue, **D**: *P. harrisoni* mucus, **E**: *P. deadalea* tissue and **F**: *P. deadalea* mucus sampled from inshore reef system of Qit’at Benaya (March 2008).


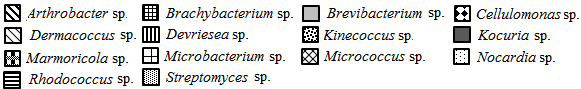


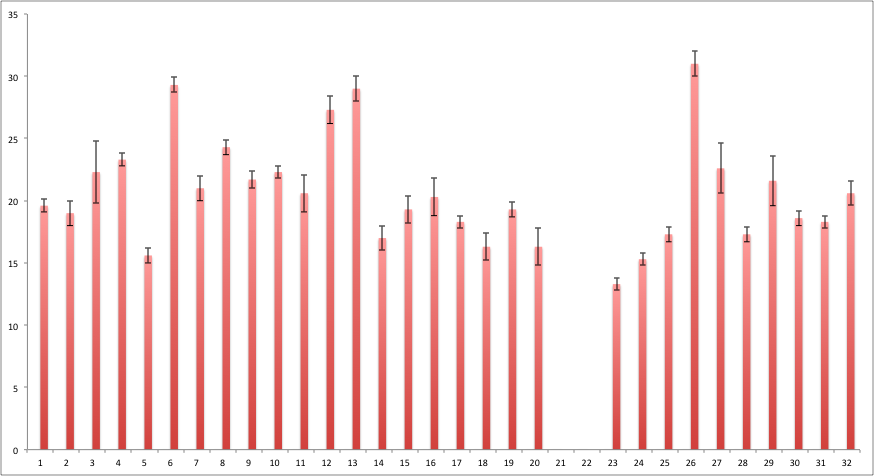


Antimicrobial activity [Inhibition zone (mm) ]

Figure S2. The inhibition zone size of 32 Streptomyces isolates on *S. aureus*. 30 out of the 32 showed strong inhibition (>15 mm inhibition zone ).

Figure S3. Neighbour-joining tree shows partial 16S rDNA sequence of *Brevibacterium* sp. found associated with Gulf coral and their nearest match from the Genbank.

Figure S4. Neighbour-joining tree shows partial 16S rDNA sequence of *Kocuria* sp. found associated with Gulf coral and their nearest match from the Genbank. The percentage of 2000 bootstrap replicate is shown below the nodes.
